# Hepatocyte Growth Factor Regulates Macrophage Transition to the M2 Phenotype and Promotes Murine Skeletal Muscle Regeneration

**DOI:** 10.3389/fphys.2019.00914

**Published:** 2019-07-25

**Authors:** Wooshik Choi, Jaeman Lee, Junghun Lee, Sang Hwan Lee, Sunyoung Kim

**Affiliations:** ^1^Department of Biological Sciences, College of Natural Sciences, Seoul National University, Seoul, South Korea; ^2^R&D Center for Innovative Medicines, ViroMed Co., Ltd, Seoul, South Korea

**Keywords:** hepatocyte growth factor, muscle regeneration, macrophage transition, AMPK, CaMKKβ

## Abstract

Hepatocyte growth factor (HGF) is well known for its role in the migration of embryonic muscle progenitors and the activation of adult muscle stem cells, yet its functions during the adult muscle regeneration process remain to be elucidated. In this study, we showed that HGF/c-met signaling was activated during muscle regeneration, and that among various infiltrated cells, the macrophage is the major cell type affected by HGF. Pharmacological inhibition of the c-met receptor by PHA-665752 increased the expression levels of pro-inflammatory (M1) macrophage markers such as IL-1β and iNOS while lowering those of pro-regenerative (M2) macrophage markers like IL-10 and TGF-β, resulting in compromised muscle repair. In Raw 264.7 cells, HGF decreased the RNA level of LPS-induced TNF-α, IL-1β, and iNOS while enhancing that of IL-10. HGF was also shown to increase the phosphorylation of AMPKα through CaMKKβ, thereby overcoming the effects of the LPS-induced deactivation of AMPKα. Transfection with specific siRNA to AMPKα diminished the effects of HGF on the LPS-induced gene expressions of M1 and M2 markers. Exogenous delivery of HGF through intramuscular injection of the HGF-expressing plasmid vector promoted the transition to M2 macrophage and facilitated muscle regeneration. Taken together, our findings suggested that HGF/c-met might play an important role in the transition of the macrophage during muscle repair, indicating the potential use of HGF as a basis for developing therapeutics for muscle degenerative diseases.

## Introduction

Resolution of tissue damage requires tight interaction between the immune system and the target tissue undergoing repair. Immune cells detect the injury, remove damaged tissues, and then promote repair mechanisms to restore tissue integrity. In addition, they strongly influence the growth and differentiation of stem cells and progenitors to repair the inflicted damage (Wynn and Vannella, 2016). Upon muscle injury, macrophages have been reported to play critical roles in this process as they occupy major cell population infiltrated in injured muscle. They are responsible for the removal of damaged myofibers and also contribute to subsequent regrowth and differentiation of muscle progenitors (Tidball, 2017). These procedures are tightly regulated by the coordinated transition of macrophage between pro-inflammatory (M1) and pro-regenerative (M2) phenotypes in the immune environment (Wang et al., 2014).

Hepatocyte growth factor (HGF) is a multifunctional protein which contains mitogenic, morphogenic, motogenic, and angiogenic activities by interacting with its cellular receptor, c-met (Nakamura and Mizuno, 2010). This interaction subsequently turns on a variety of signaling pathways depending on cell types. HGF has been shown to play important roles in the regeneration process of various tissues by stimulating the proliferation and migration of respective progenitor cells (Kawaida et al., 1994; Huh et al., 2004; Wu et al., 2004; Watanabe et al., 2005). For example, HGF has been implicated in both skeletal muscle development and its regeneration after injury (Sisson et al., 2009).

The interaction between HGF and c-met is required for migration of myogenic progenitors into the limb buds during embryogenesis (Dietrich et al., 1999). Upon muscle injury, HGF activates muscle stem cells that reside in muscle fiber, rendering them to make a myogenic commitment (Jennische et al., 1993; Allen et al., 1995; Tatsumi et al., 1998; Sheehan et al., 2000). Exogenously added recombinant HGF protein was reported to increase myoblast proliferation while inhibiting differentiation, resulting in delayed regeneration of damaged muscle (Miller et al., 2000). However, recent studies have shown that HGF activated by urokinase plasminogen activator (uPA) promotes muscle regeneration (Sisson et al., 2009), and its receptor, c-met, is responsible for the transition of quiescent muscle stem cells into GAlert, a cellular state in which they have an increased ability to participate in tissue repair (Rodgers et al., 2014). Thus, the role of HGF in muscle regeneration remains to be clarified. In particular, although the immune system plays an instrumental role in the muscle regeneration process, the effect of HGF on immune cells infiltrated in injured muscle tissue is not yet clearly understood.

Here, we report the role of HGF in the transition of infiltrated macrophages during muscle regeneration. HGF expression was upregulated following muscle injury. When mice were treated with PHA-665752, an inhibitor of the c-met receptor, muscle regeneration was delayed. Consistently, the population of M1 and M2 macrophages during muscle regeneration was deregulated. HGF overexpression by intramuscular (i.m.) injection of plasmid expression vector facilitated muscle regeneration. Data from experiments involving Raw 264.7 cells indicated that HGF might regulate the transition of macrophage to the M2 phenotype through CaMKKβ-AMPK signaling. Taken together, our data suggested that HGF might be used as a platform for developing therapeutic agents to treat diseases associated with defects in muscle regeneration.

## Materials and Methods

### Animal Cares

Ten-week-old male C57BL/6 mice were purchased from Orient Bio Inc. (Seongnam, Korea) for animal studies. Mice were housed at 24°C with a 12 h light-dark cycle. All experiments were performed in compliance with the guideline set by the International Animal Care and Use Committee at Seoul National University.

### Surgical Procedures

All surgical protocols were approved by the International Animal Care and Use Committee at Seoul National University. For muscle injury, ten-week-old male C57BL/6 mice were anesthetized with isoflurane, and the TAs were injected with 50 μl CTX (Latoxan, Valence, France), diluted to 10 μM in phosphate buffered saline (PBS). Sham treatment was performed by following the same procedure except injecting TAs with PBS. PHA-665752 (Tocris Bioscience, MO), a c-met inhibitor, was dissolved in DMSO (Sigma Aldrich, MO) and i.p. administered in each mouse on a daily basis with a dose of 20 mg/kg. For i.m. injection, 0.3 mm needle size, 0.5 ml insulin syringe (BD, NJ) was used. pCK or pCK-HGF-X7 plasmid expression vector was dissolved in 50 μl PBS (2 μg/μl). The injection procedure was performed by injecting the needle parallel to the tibia and then delivering plasmid into the middle of the TA.

### Immunohistochemistry

Immunohistochemical analyses were performed as previously described (Ahn et al., 2014). Briefly, TAs were isolated and fixed in 4% paraformaldehyde in PBS and cryo-sectioned to 6 μm thickness. Sections were washed in 0.1 M PBS (pH 7.4) twice, then blocked for 1 h with PBS containing 5% fetal bovine serum (Corning, NY), 5% donkey serum (Jackson ImmunoResearch Laboratories, PA), 2% BSA (Sigma Aldrich, MA) and 0.1% Triton X-100 (Sigma Aldrich, MA). Samples were incubated with primary antibodies diluted in blocking buffer overnight at 4°C. Sections were washed 4 times in PBS and incubated for 1 h at room temperature with secondary antibodies (Invitrogen, CA) diluted in PBS. Immunostained samples were further washed 6 times and counterstained with DAPI (Sigma Aldrich, MA) for nuclear staining. The fluorescence images were obtained using a Zeiss LSM 700 confocal microscope (Zeiss, Oberkochen, Germany).

### Hematoxylin and Eosin Staining and Morphometric Analysis

TAs were fixed in 10% normalized buffered formalin (Sigma Aldrich, MA) and dehydrated with a gradient series of ethanol from 70 to 100%. Samples were embedded in the paraffin block and sectioned to 6 μm thickness. A paraffin section of the TA was stained by hematoxylin and eosin, and the morphology of each cross-section was analyzed by Image J software (National Institutes of Health, MD). More than 300 myofibers were assessed from four individual mice in each group.

### RNA Isolation and RT-qPCR

TAs were prepared and mechanistically homogenized using Bullet Blender Storm (Next Advance, NY), and total RNA was extracted from homogenized TA or cultured cells with RNAiso (Takara, Kusatsu, Japan) following the manufacturer’s instructions. One microgram of RNA was converted to cDNA using oligo dT primers (Qiagen, Hilden, Germany) and Reverse Transcriptase XL (AMV) (Takara, Kusatsu, Japan). Gene expression was assessed using quantitative real-time PCR with Thermal Cycler Dice Real Time System TP800 (Takara, Kusatsu, Japan) and SYBR Premix Ex Taq (Takara, Kusatsu, Japan). Primers used in this study are listed in Supplementary Table S1A.

### ELISA

TAs were prepared and mechanistically homogenized using Bullet Blender Storm (Next Advance, NY), and total proteins were extracted in RIPA lysis buffer (Sigma Aldrich, MO) containing a protease inhibitor (Roche, Basel, Switzerland), phosphatase inhibitor (Roche, Basel, Switzerland), and PMSF (Sigma Aldrich, MO). Samples were centrifuged at 12,000 rpm for 15 min at 4°C, and the supernatants containing total protein were subjected to mHGF ELISA (R&D systems, MN) following the manufacturer’s protocol.

### Western Blot

For immunoblotting, TAs or cultured cells were prepared and homogenized in RIPA lysis buffer (Sigma Aldrich, MO) containing a protease inhibitor (Roche, Basel, Switzerland) and phosphatase inhibitor (Roche, Basel, Switzerland) using Bullet Blender Storm (Next Advance, NY). Equal amounts of protein were then separated by 10% SDS-polyacrylamide gel and electrophoretically transferred to polyvinylidene fluoride membranes (Millipore, MA). The membranes were blocked with 5% BSA (Gibco, MA) in TBST (1 M Tris-HCl, pH 7.4, 0.9% NaCl and 0.1% Tween-20) for 1 h and probed with antibodies diluted in 3% BSA blocking solution overnight at 4°C. Membranes were then incubated with HRP-conjugated anti-mouse or anti-rabbit IgG (1:100,000; Sigma Aldrich, MO) for 1 h, and the protein bands were visualized with the enhanced chemiluminescence system (Millipore, MA). Antibodies used in this study are listed in Supplementary Table S1B.

### Cell Culture and Reagents

Raw 264.7 cells (American Type Culture Collection, VA) were grown in DMEM (Welgene, Gyeongsan, Korea) supplemented with 10% FBS (Corning, NY) and antibiotics [100 U/ml penicillin and 100 μg/ml streptomycin (Sigma Aldrich, MO)]. Recombinant human HGF protein (R&D systems, MN) and LPS (Sigma Aldrich, MO) were used at appropriate concentrations. STO-609 (CaMKKβ inhibitor, Tocris Bioscience, MO) was used at 5 or 20 μM for experiments.

### siRNA Transfection

Raw 264.7 cells were transfected with siRNA specific to AMPKα or scramble siRNA (Santa Cruz Biotechnology, TX) using RNAiMAX (ThermoFisher Scientific, MA) according to the manufacturer’s protocol. Briefly, Raw 264.7 cells were plated at 1 × 10^6^ cells per well in 6-well culture plates. Twenty-four hours later, 25 pmol (2.5 μl, 10 μM) of AMPKα or scramble siRNA was diluted into 125 μl of Opti-MEM (Gibco, MA), and 5 μl of RNAiMAX was diluted in 125 μl of Opti-MEM. Diluted siRNA and RNAiMAX were then combined and incubated at room temperature for 5 min. Subsequently, 250 μl of the siRNA-RNAiMAX mixtures were added to each well of a 6-well plate. Twenty-four hours after transfection, cells were subjected to the analysis. Knock-down efficiency was evaluated by western blot using antibodies against AMPKα (Cell Signaling Technology, MA).

### Statistical Analysis

All values are represented as mean ± SEM from two or more independent experiments. Statistical significance was determined using unpaired student’s *t* test or one way ANOVA followed by Bonferroni’s multiple comparison tests, provided by the GraphPad Prism 7 (GraphPad, CA) software.

## Results

### HGF/c-Met Signaling Was Upregulated During Muscle Regeneration

**Figure 1:**
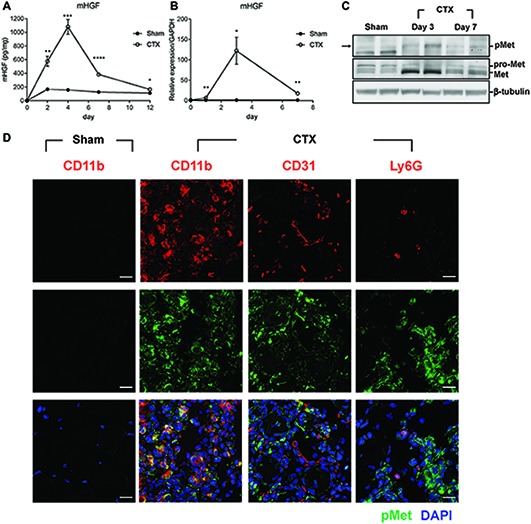
HGF/c-met signaling is activated during muscle regeneration. **(A)** Expression kinetics of HGF protein during muscle injury by CTX and regeneration. The muscle was isolated at 2, 4, 7, and 12 days after CTX injection, and total proteins were analyzed by ELISA to measure the protein levels of HGF. ^*^p < 0.05, ^**^p < 0.01, ^***^p < 0.001, ^****^p < 0.0001 versus sham-treated muscles (unpaired student’s *t* test), *n* = 4 per group. **(B)** Expression kinetics of the RNA levels of HGF during muscle injury and regeneration. RNA was prepared from TAs 1, 3, and 7 days after CTX injection followed by RT-qPCR, ^*^p < 0.05, ^**^p < 0.01 versus sham-treated muscles (unpaired student’s *t* test), *n* = 4 per group. The values were normalized to glyceraldehyde-3-phosphate dehydrogenase (GAPDH). **(C)** Expression kinetics of total and phosphorylated c-met proteins in CTX-injured TAs. Muscles were isolated at 3 and 7 days postinjury, and total proteins were prepared followed by western blot using specific antibodies to total or phosphorylated c-met. β-tubulin was used as a loading control. Each lane represents a sample from an individual mouse. Two representative mice are shown here. Two independent experiments were performed (with a total of four mice), and similar results were obtained. Arrow indicates the protein of interest in blots. **(D)** Identification of cell types expressing c-met. CTX-injured TA was isolated 3 days postinjury and subjected to immunofluorescence assay using antibodies to CD11b for macrophages, CD31 for endothelial cells, Ly6G for neutrophils (all red), and phosphorylated c-met (green). Nuclei were counterstained with DAPI (blue). *n* = 4 per group. Scale bars, 20 μm. All data are presented as mean ± SEM. See also Supplementary Figure S1.

To investigate the possible involvement of HGF during muscle regeneration, cardiotoxin (CTX)-induced muscle injury model was used. Injection of CTX provides homogenous damage to the whole muscle and induces the infiltration of various immune cells including monocytes and macrophages into the regenerating muscle until the repair is completed (Arnold et al., 2007). Tibialis anterior (TA) muscle of a 10-week-old C57BL/6 mouse was injected with CTX or PBS (sham), and total proteins were prepared from the TA at appropriate time points followed by ELISA. The basal level of the HGF protein in the sham (PBS-injected) muscle was maintained at 120–160 pg/mg of total cellular protein in the TA (Figure 1A). After muscle injury, the level of the HGF protein in the injured side was gradually increased, reaching a peak at approximately 1.1 ng mg of total cellular protein at day 4, and then steadily decreased before returning to the sham level at day 12. A similar magnitude of HGF RNA induction was observed during muscle regeneration as measured by RT-qPCR (Figure 1B). These data suggested that HGF expression was highly induced after muscle injury at both RNA and protein levels.

c-met is the only known receptor for HGF. When HGF is expressed, the c-met protein becomes phosphorylated to be activated. Therefore, the level and content of the c-met protein was analyzed after muscle injury. Total proteins were prepared from the TA followed by western blot using antibodies to total c-met or phosphorylated form (Figure 1C). During muscle regeneration, the level of total c-met protein rapidly increased and the phosphorylated form of the c-met protein was also upregulated in the damaged muscle.

To identify the cell types that express c-met, cells known to be infiltrated in the injured muscle were analyzed by IHC using antibodies to phosphorylated c-met, CD11b for macrophages, CD31 for endothelial cells, and Ly6G for neutrophils, 3 days after muscle injury. As shown in Figure 1D, the major cell type containing activated c-met was macrophages. In sham-treated muscle, there were no cells expressing phosphorylated c-met (Supplementary Figure S1A). Since macrophages are known to be a primary source of HGF after injury (Sisson et al., 2009), these data indicated that in an injured muscle, HGF might act on macrophage in an autocrine manner.

### Inhibition of c-Met Signaling Delayed Muscle Regeneration

**Figure 2:**
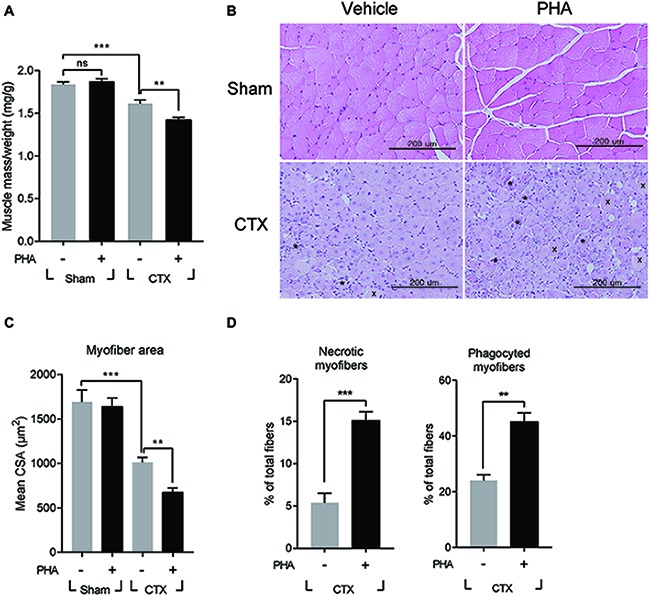
Effects of c-met inhibitor PHA-665752 on muscle regeneration. After CTX injury, mice were i.p. injected with 20 mg/kg of PHA-665752 on a daily basis until sacrificed. CTX injured TAs were analyzed 7 days postinjury. **(A)** Effect on muscle weight. Muscle mass was normalized with the weight of mice. PHA, PHA-665752. ns, not significant, ^**^p < 0.01, ^***^p < 0.001 (one-way ANOVA), *n* = 4 per group. **(B)** H&E staining of regenerating muscle. Crosses (x) and asterisks (*) indicate necrotic and phagocyted myofibers, respectively. Scale bars, 200 μm. **(C)** Effect on cross-sectional areas of muscle fibers. Mean value of area sizes is indicated in the graph. ^**^p < 0.01, ^***^p < 0.001 (one-way ANOVA), *n* = 4 per group. **(D)** Quantification of necrotic or phagocyted fibers expressed as a percentage of total myofibers. At least 300 muscle fiber areas were counted per sample. ^**^p < 0.01, ^***^p < 0.001 (unpaired student’s *t* test), *n* = 4 per group. All data are presented as mean ± SEM. See also Supplementary Figure S2.

It was tested whether increased expression of HGF would contribute to or inhibit muscle regeneration using an inhibitor specific to the c-met receptor, PHA-665752. After CTX injection, mice were intraperitoneally (i.p.) injected with PHA-665752 on a daily basis. We have previously reported that i.p. injection of PHA-665752 could effectively inhibit the phosphorylation of c-met *in vivo* including in the muscle tissue (Choi et al., 2018; Ko et al., 2018). Treatment with PHA-665752 also suppressed c-met phosphorylation in macrophages infiltrating the injured muscle (Supplementary Figure S2A). Seven days after CTX injection, TA mass from vehicle (DMSO)-treated animals was found to be reduced by 12% from 38.7 ± 0.7 to 33.9 ± 0.9 mg, compared to that of the sham-operated group, while PHA-665752 treated mice showed a larger reduction, by 19% (Figure 2A). The skeletal muscle cross-section was analyzed by hematoxylin and eosin (H&E) staining of the TA (Figure 2B). In vehicle-treated mice, muscle fiber size was decreased by 40 ± 3% from 1,690 ± 134 to 1,014 ± 55 μm^2^ compared with that of the sham-operated animals. In PHA-665752 treated mice, it was further reduced, by 60 ± 3% compared to the sham-operated group (Figure 2C). In addition, the number of necrotic or damaged phagocytic fibers in PHA-treated animal was significantly higher in comparison to the vehicle-treated group (Figure 2D). These data indicated that the inhibition of c-met signaling could delay the restoration of muscle mass and muscle fiber regeneration, suggesting that HGF might play a positive role(s) in muscle repair.

**Figure 3:**
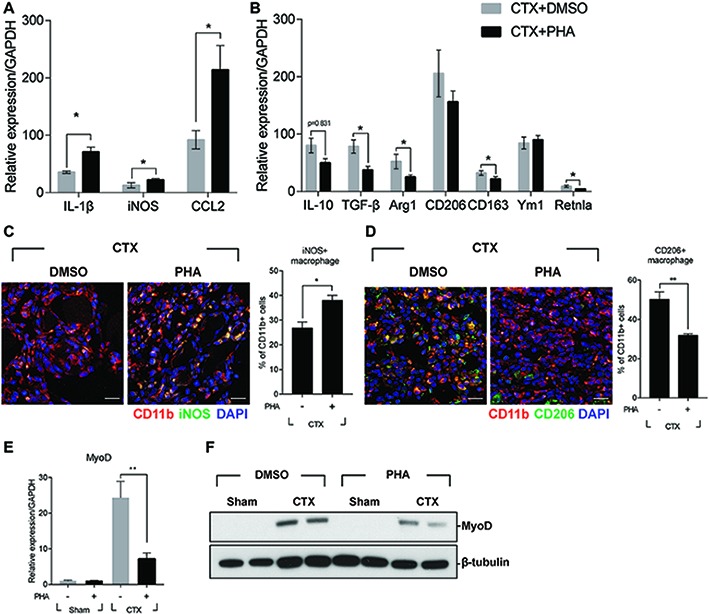
Effects of c-met inhibitor PHA-665752 on macrophage population infiltrated in the muscle. After CTX injury, mice were i.p. injected with 20 mg/kg of PHA-665752 on a daily basis until sacrificed. **(A)** Effects on the RNA levels of M1 markers (iNOS, IL-1β, and CCL2). **(B)** Effects on the RNA levels of M2 markers (IL-10, TGF-β, Arg1, CD206, CD163, Ym1, and Retnla). The RNA level of these genes isolated from TAs 3 days after injury was determined by RT-qPCR. The relative expression level of sham-operated, vehicle-treated mice is presented as 1. **(C)** Effect on iNOS-positive macrophages and **(D)** CD206-positive macrophages. CTX-injured TA was isolated 3 days postinjury and subjected to immunofluorescence assay using antibodies to CD11b (red) and iNOS or CD206 (green). Nuclei were counterstained with DAPI (blue). Percentage of iNOS+ or CD206+ macrophages was indicated in the graph. ^*^p < 0.05, ^**^p < 0.01 (unpaired student’s *t* test), *n* = 4 per group. Scale bars, 20 μm. **(E)** Effects on MyoD RNA. ^*^p < 0.05 (unpaired student’s *t* test), *n* = 4 per group. **(F)** Effects on MyoD protein. Two representative results are shown here, *n* = 4 per group. All data are presented as mean ± SEM.

It was reported that defects in the process of removing damaged muscle fibers are associated with the improper transition of macrophage from M1 toward M2 phenotype (Mounier et al., 2013). Therefore, the effect of PHA-665752 on the expression of M1 and M2 macrophage markers were tested. Three days after muscle injury, RNAs were isolated from TAs of mice when the RNA level of M1 and M2 macrophage markers were greatly induced. In animals treated with PHA-665752, the level of IL-1β, iNOS, and CCL2 (M1 markers) further increased (Figure 3A), while that of IL-10, TGF-β, arginase 1 (Arg1), CD163, and resistin-like alpha (Retnla) (M2 markers) was reduced, in comparison to the vehicle-treated group (Figure 3B).

To identify whether the regulation of M1 and M2 marker genes was caused by the changes in the population of macrophage, injured muscle was analyzed by immunofluorescence assay using antibodies to iNOS for M1 macrophages, and CD206 for M2 macrophages. Three days after muscle injury, the number of iNOS-positive macrophages was highly increased and was further enhanced in animals treated with PHA-665752 (Figure 3C). The number of CD206-positive macrophages was also increased upon injury, but PHA-665752 treatment significantly reduced it (Figure 3D). These results indicated that the HGF/c-met signaling pathway might be involved in the transition of macrophages to an appropriate type during muscle regeneration.

Muscle regeneration is controlled by spatiotemporal regulation of various myogenic regulatory factors (MRFs) during which infiltrated immune cells play key roles (Di Marco et al., 2005; Tidball and Villalta, 2010). Therefore, the expression of myoD, the key player in the regulation of myogenic commitment, was tested. Three days after muscle injury, the RNA level of myoD was markedly increased in CTX-injured TA, but treatment with PHA-665752 inhibited injury-induced myoD expression (Figure 3E). The protein level of myoD showed a similar pattern (Figure 3F). Taken together, our results implied that HGF/c-met signaling would act to regulate macrophage M1-M2 transition, thereby contributing to regeneration of injured muscle.

### HGF Promotes M2 Macrophage Transition *via* CaMKKβ-AMPK Signaling

**Figure 4:**
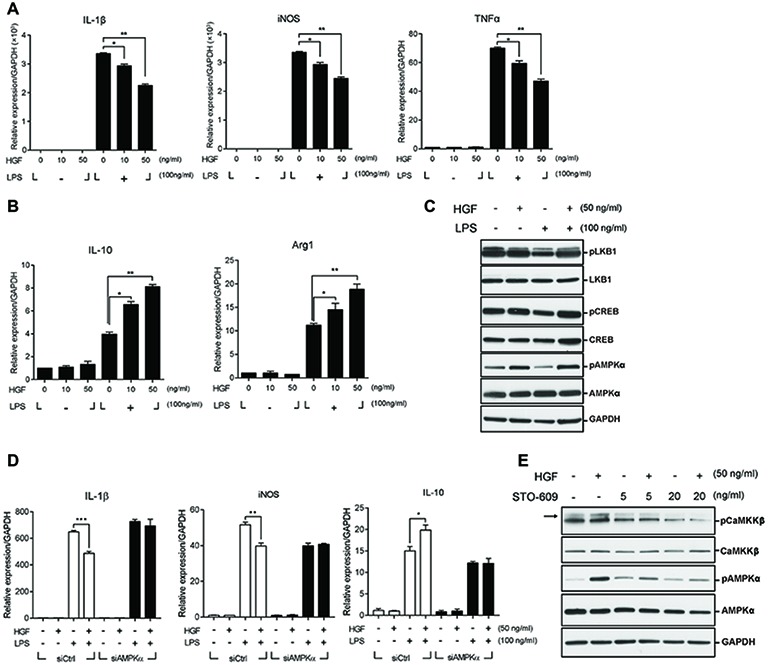
Roles of CaMKKβ-AMPKα on HGF-mediated control of the expression of M1 and M2 markers in Raw 264.7 cells. Raw 264.7 cells were cultured in the presence or absence of LPS and recombinant HGF proteins. Total RNA and proteins were prepared and analyzed by RT-qPCR and western blot, respectively. **(A)** Effects of HGF on the RNA levels of M1 markers (IL-1β, iNOS, and TNFα). ^*^p < 0.05, ^**^p < 0.01 (unpaired student’s *t* test), *n* = 3 per group. **(B)** Effects of HGF on RNA levels of M2 markers (IL-10 and Arg1). Values were normalized to GAPDH. ^*^p < 0.05, ^**^p < 0.01 (unpaired student’s *t* test), *n* = 3 per group. **(C)** Effects of HGF on signaling pathways related to macrophage polarization. **(D)** Effects of AMPKα knockdown on HGF-mediated regulation of the RNA level of IL-1β, iNOS, and IL-10, marker genes of M1 and M2. Raw 264.7 cells were transfected with AMPKα or control siRNAs, and then treated with LPS and HGF. Values were normalized to GAPDH. ^*^p < 0.05, ^**^p < 0.01, ^***^p < 0.001 (unpaired student’s *t* test), *n* = 3 per group. **(E)** Effects of CaMKKβ inhibitor, STO-609, on the HGF-mediated phosphorylation of AMPKα. Arrow indicates the protein of interest in blots. In western blot hybridization, GAPDH was used as a loading control. All data are presented as mean ± SEM. See also Supplementary Figure S2 and S3.

To understand the mechanism(s) underlying the effect of HGF on macrophage transition at the molecular and cellular levels, Raw 264.7 cells, a murine macrophage line, was used. Lipopolysaccharide (LPS) was used to activate these cells to the pro-inflammatory phenotype (M1) in order to mimic *in vivo* muscle injury. Cells were treated with various concentrations of the recombinant human HGF (hHGF) protein in the presence of 100 ng/ml of LPS for 24 h. When Raw 264.7 cells were treated with LPS only, the expression level of M1 markers (IL-1β, iNOS, and TNFα) was greatly induced compared to the untreated control. Co-treatment with HGF inhibited an LPS-mediated increase in the level of M1 maker genes in a dose-dependent manner resulting in an approximate 40% decrease compared to the LPS only group (Figure 4A). On the other hand, LPS stimulation increased the expression level of IL-10 and Arg1, representative M2 marker genes, and HGF treatment further increased it in a dose-dependent manner (Figure 4B), suggesting that HGF might indeed control macrophage M2 transition.

The transition of macrophages from M1 to M2 during muscle regeneration is known to be controlled by two different pathways; CREB-C/EBPβ pathway (Ruffell et al., 2009) or AMPK signaling (Mounier et al., 2013). HGF had little or no effect on the former, since treatment with HGF did not affect the phosphorylation of CREB and the expression of C/EBPβ (Figure 4C, Supplementary Figure S3B). To test whether HGF regulates the macrophage transition through AMPK, Raw 264.7 cells were treated with 50 ng/ml of the hHGF protein and 100 ng/ml LPS for 30 min, followed by western blot, using antibodies to total or phosphorylated AMPKα. LPS stimulation decreased the level of phosphorylated AMPKα as previously reported (Sag et al., 2008), but the presence of HGF restored the phosphorylation of AMPKα to the normal level. The level of total AMPKα remained unchanged in all conditions (Figure 4C).

To test if AMPK is involved in the effects of HGF on inflammatory cytokines production, Raw 264.7 cells were transfected with siRNA against AMPKα followed by treatment with LPS and HGF. The protein level of AMPKα was highly reduced by siRNA transfection (Supplementary Figure S3A). Raw 264.7 cells transfected with siAMPKα had no effect on the HGF-mediated control of M1 and M2 marker gene expressions (Figure 4D). These results indicated that HGF controlled the expression of M1 markers (IL-1β and iNOS) and IL-10 by upregulating AMPK phosphorylation.

AMPK phosphorylation is known to be regulated by upstream effectors such as LKB1 and CaMKKβ (Carling et al., 2008). It was tested as to which upstream effectors would be involved in the phosphorylation of AMPK mediated by HGF. LKB1 did not seem to play a role as HGF did not induce the phosphorylation of LKB1 in Raw 264.7 cells (Figure 4C). However, treatment with HGF induced the phosphorylation of CaMKKβ and treatment with STO-609, CaMKKβ inhibitor, lowered the level of HGF-mediated phosphorylation of AMPKα (Figure 4E). Therefore, in Raw 264.7 cells, CaMKKβ, but not LKB1, appeared to act as an upstream effector of HGF-mediated control of AMPK.

Finally, it was investigated whether the phosphorylation of AMPK was regulated by HGF during skeletal muscle regeneration. At day 3 postinjury, TAs were isolated and subjected to western blot and immunofluorescence. The level of phosphorylated AMPKα was highly increased in CTX-injured TA, and treatment with PHA-665752 had no significant effect (Supplementary Figure S2B). When the phosphorylation of AMPK was measured *in situ* specifically in macrophages, however, a completely different picture emerged; PHA-665752 treatment significantly reduced the injury-mediated phosphorylation of AMPK in macrophages (Supplementary Figure S2C). Taken together, these data suggested that HGF/c-met pathway regulate M2 macrophage transition by regulating CAMKKβ and AMPK.

### Exogenous Delivery of HGF Facilitated Muscle Regeneration

Based on the above results indicating a positive role(s) of HGF in macrophage transition during muscle regeneration, we tested the effects of the exogenous addition of HGF in the same CTX muscle injury model. Since HGF has a very short half-life, less than 5 min in serum (Kawaida et al., 1994), we delivered HGF in the form of a plasmid DNA expression vector. pCK-HGF-X7 (or VM202) is a plasmid designed to produce two isoforms of human HGF, HGF723 (or dHGF), and HGF728 (or cHGF), at high levels *in vivo* (Cho et al., 2008; Pyun et al., 2010; Hahn et al., 2011), and it has been known to work in a variety of clinical studies and animal models (Cho et al., 2008; Pyun et al., 2010; Hahn et al., 2011; Kessler et al., 2015; Kibbe et al., 2016). The *in vivo* protein expression kinetics of this plasmid in the muscle have been well established previously (Supplementary Figure S4A) (Cho et al., 2008; Pyun et al., 2010; Hahn et al., 2011; Nho et al., 2018).

**Figure 5:**
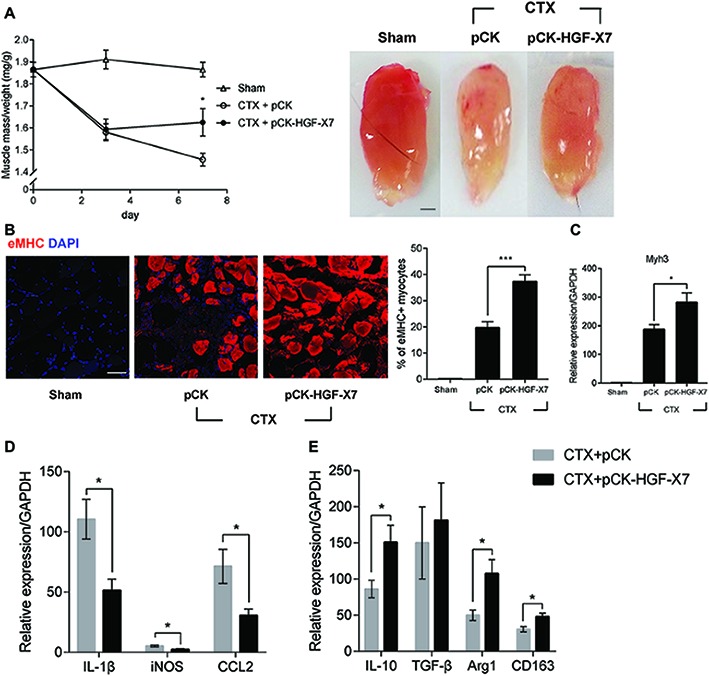
Effects of HGF overexpression by intramuscular injection of HGF expressing plasmid on muscle regeneration. pCK or pCK-HGF-X7 was i.m. injected 3 days prior to the CTX injection. TAs were prepared at appropriate times after injury. **(A)** Effect on TA weight. Representative TAs from 7 days postinjury are shown in the photos. ^*^p < 0.05 versus CTX + pCK group (one-way ANOVA), *n* = 4 per group. Scale bar, 1 mm. **(B)** Effects on cross-sectional areas of regenerating fibers. CTX-injured TA was isolated 3 days postinjury and subjected to immunofluorescence assay using antibodies to eMHC (red) for regenerating myofibers. Percentage of eMHC+ myocytes was indicated in the graph. ^***^p < 0.001 (one-way ANOVA), *n* = 4 per group. Scale bar, 50 μm. **(C–E)** Three days after injury, the TA was isolated, and total RNAs were analyzed by RT-qPCR. **(C)** Effects on the expression of Myh3. ^*^p < 0.05 (one-way ANOVA), *n* = 4 per group. **(D)** Effects on the RNA levels of M1 markers (IL-1β, iNOS, and CCL2). ^*^p < 0.05 (one-way ANOVA), *n* = 4 per group. **(E)** Effects on RNA levels of M2 markers (IL-10, TGF-β, Arg1, and CD163). ^*^p < 0.05 (one-way ANOVA), *n* = 4 per group. Values were normalized to GAPDH. All data are presented as mean ± SEM. See also Supplementary Figure S4.

Three days prior to the muscle injury, 100 μg of pCK-HGF-X7 or pCK control vector lacking the HGF sequence was i.m. administered into the TA, and muscle injury was introduced by injecting CTX to the TA of a 10-week-old C57BL/6 mouse. The TA was isolated, and muscle mass was quantitated at different time points after muscle injury. As shown in Figure 5A, in CTX-injured mice injected with pCK control vector, muscle mass was decreased by 18 and 22% at days 3 and 7, respectively. When mice were injected with pCK-HGF-X7, the restoration of muscle weight was facilitated, to 17 and 13% decrease, compared to the control at days 3 and 7, respectively.

The regenerating muscle fibers were analyzed by immunostaining embryonic myosin heavy chain (eMHC), 3 days after muscle injury. In pCK-treated animals, the percentage of eMHC+ myocytes was about 20%, while it increased to 37% when treated with pCK-HGF-X7 (Figure 5B). Similar patterns were observed when the RNA level of eMHC (Myh3) was measured by qRT-PCR (Figure 5C). Overall, our data showed that the exogenous addition of HGF, delivered in the form of plasmid expression vector, could facilitate muscle regeneration.

The effects of i.m. injection of pCK-HGF-X7 on M1 and M2 markers were also measured. Muscle injury was induced, and pCK or pCK-HGF-X7 was i.m. injected into the TA. Three days after muscle injury, TA was isolated, and the RNA level of M1 and M2 markers were measured using RT-qPCR. The level of M1 markers, IL1β, iNOS, and CCL2 was highly increased after muscle injury, but pCK-HGF-X7 injection reduced the expression of these genes (Figure 5D). The level of M2 markers, IL-10, Arg1, and CD163, also increased upon muscle injury and further increased in animals treated with PCK-HGF-X7 (Figure 5E). These data strongly indicated that HGF overexpression by gene transfer technology could trigger transition of macrophages to the M2 phenotype to promote muscle regeneration.

## Discussion

In this report, we demonstrated that HGF/c-met signaling plays a key role in the regulation of macrophage transition during muscle regeneration after necrotic injury. The HGF/c-met signaling was highly activated after muscle damage, and the macrophage was the major cell type affected by HGF among cells that infiltrated the muscle. Treating CTX-injured mice with PHA-665752, a specific inhibitor for c-met, deregulated the population of macrophages and delayed muscle regeneration. Exogenous supply of the HGF protein to the affected region through i.m. injection of a highly efficient plasmid expression vector promoted the transition of macrophage to the M2 phenotype and facilitated muscle regeneration. Data from Raw 264.7 cells showed that HGF controls macrophage transition *via* CaMKKβ-AMPK signaling. Taken together, HGF/c-met signaling plays a key role in the transition of the macrophage infiltrated during muscle regeneration.

HGF is secreted by a variety of cell types in cases of muscle injury. Relevant to our work is that fact that, macrophages are known to be the primary source of HGF during muscle regeneration (Sisson et al., 2009). Consistent with previous reports, we found that the kinetics of the RNA and protein levels of HGF paralleled the curve of macrophagic appearance in regenerating muscle (Mounier et al., 2013). However, the underlying mechanism by which macrophages produce HGF has not yet been understood. One possibility is that HGF may be secreted when these cells are exposed to apoptotic neutrophils. It was previously reported that when macrophages encounter apoptotic cells, HGF is produced *via* a RhoA-dependent signal *in vitro* (Park et al., 2011). Interestingly, our study showed that the expression level of HGF peaked at 3–4 days postinjury, and this is the time when neutrophils induce an early immune response and became apoptotic and cleared by macrophages (Arnold et al., 2007; Nguyen et al., 2011). It remains to be elucidated whether HGF expression in macrophages would indeed be regulated by their exposure to apoptotic neutrophils *in vivo*.

We found that HGF regulated phosphorylation of AMPK through the CaMKKβ upstream regulator. Since HGF/c-met signaling promotes the influx of calcium ions into a variety of cells, it is possible that calcium-dependent kinases are activated (Baffy et al., 1992; Tyndall et al., 2007; Gomes et al., 2008). In our study involving Raw 264.7 cells, another well-known upstream regulator, LKB1, was not phosphorylated by HGF. This is different from the previous study showing that HGF regulates the phosphorylation of AMPK through LKB1 in primary hepatocytes (Vázquez Chantada et al., 2009). In cases of muscle injury, however, it was previously reported that mice lacking LKB1 in their myeloid cells did not show any significant defects during muscle regeneration (Mounier et al., 2013). Therefore, LKB1 in macrophages may play a negligible role in the HGF/AMPK-mediated regulation of macrophage transition during muscle repair. These data imply that HGF might regulate the phosphorylation of AMPK through a different pathway depending on cell type.

In this study, we focused on the role of HGF/c-met signaling on macrophages during muscle regeneration, as they are the major cell type infiltrated after muscle injury. We found that HGF regulated the transition of the macrophage phenotype to promote muscle repair; while others reported that when c-met is knocked out specifically in muscle stem cells, mice showed delayed stem cell activation, decreased myoblast motility, abnormalities in myocyte fusion, and impaired muscle regeneration (Webster and Fan, 2013). These results suggest that HGF/c-met signaling could act on various cell types, including muscle progenitors and invading immune cells, to coordinate muscle repair during muscle regeneration. Also, HGF may be able to produce multiple positive effects in various cells during muscle regeneration.

In summary, HGF/c-met signaling appears to play a role(s) in muscle repair by promoting macrophage transition to the M2 phenotype. Various muscle diseases, including muscular dystrophy or myositis, are accompanied by a high inflammatory burden, leading to tissue lysis and compromising of the regeneration process. Currently, no drug can discriminate between the different phenotypes of the macrophage. Without such specificity, there is a chance of indiscriminate macrophage depletion, which can lead to undesirable systemic side effects (Balaban et al., 2005). Given the safety and efficacy records of pCK-HGF-X7 (VM202) observed in several clinical studies for other indications, further studies are warranted to investigate the possibility of using HGF, and in particular the plasmid DNA vector expressing HGF, for various muscle degenerative diseases.

## Author Contributions

WC designed the study, performed the experiments, analyzed the data, and wrote the manuscript. JaL, JuL, and SL conducted the experiments. SK designed the study and wrote the manuscript.

### Conflict of Interest Statement

JuL and SK are employees or shareholders of ViroMed Co., Ltd., whose plasmid DNA (pCK-HGF-X7) was used in this work.

The remaining authors declare that the research was conducted in the absence of any commercial or financial relationships that could be construed as a potential conflict of interest.
