# Supplementary material for: Hepatocyte Growth Factor Regulates Macrophage Transition to the M2 Phenotype and Promotes Murine Skeletal Muscle Regeneration
Source: Front Physiol. 2019 Jul 25;10:914. doi: 10.3389/fphys.2019.00914 (PMC6672745; doi:10.3389/fphys.2019.00914)
Supplement: Supplementary file 1 [file Data_Sheet_1.pdf]

Figure S1, related to Figure 1

A

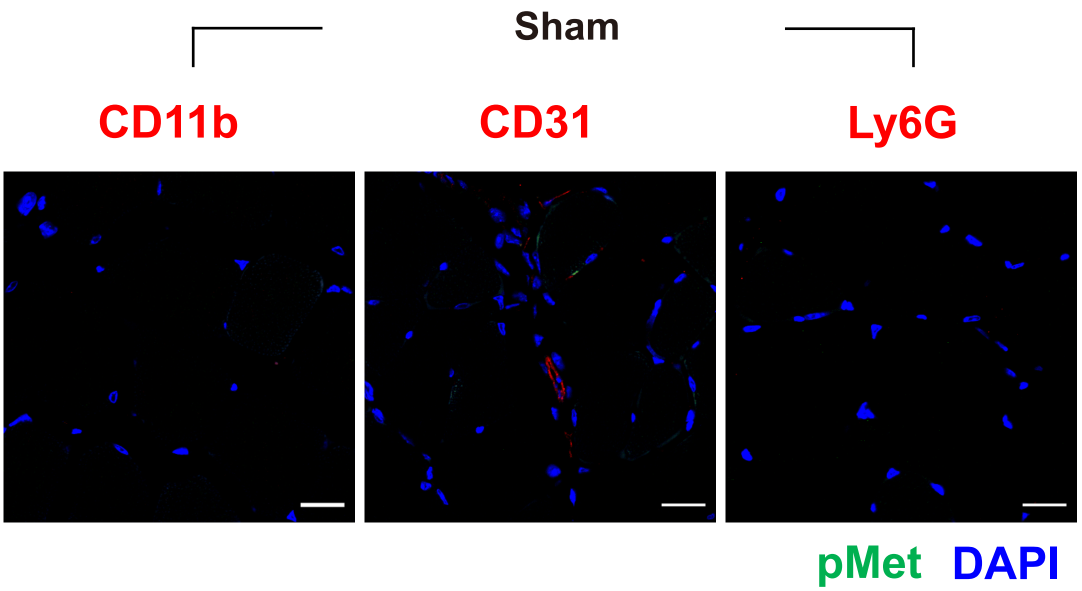

Figure S1. Baseline staining of macrophages, endothelial cells, and neutrophils in non-injured muscle.
(A) Sham (PBS)-treated TA was isolated 3 days after injection and subjected to immunofluorescence assay using antibodies to CD11b for macrophages, CD31 for endothelial cells, Ly6G for neutrophils (all red), and phosphorylated c-met (green). Nuclei were counterstained with DAPI (blue). n=4 per group. Scale bars, 20 µm.

Figure S2, related to Figure 2 and 4

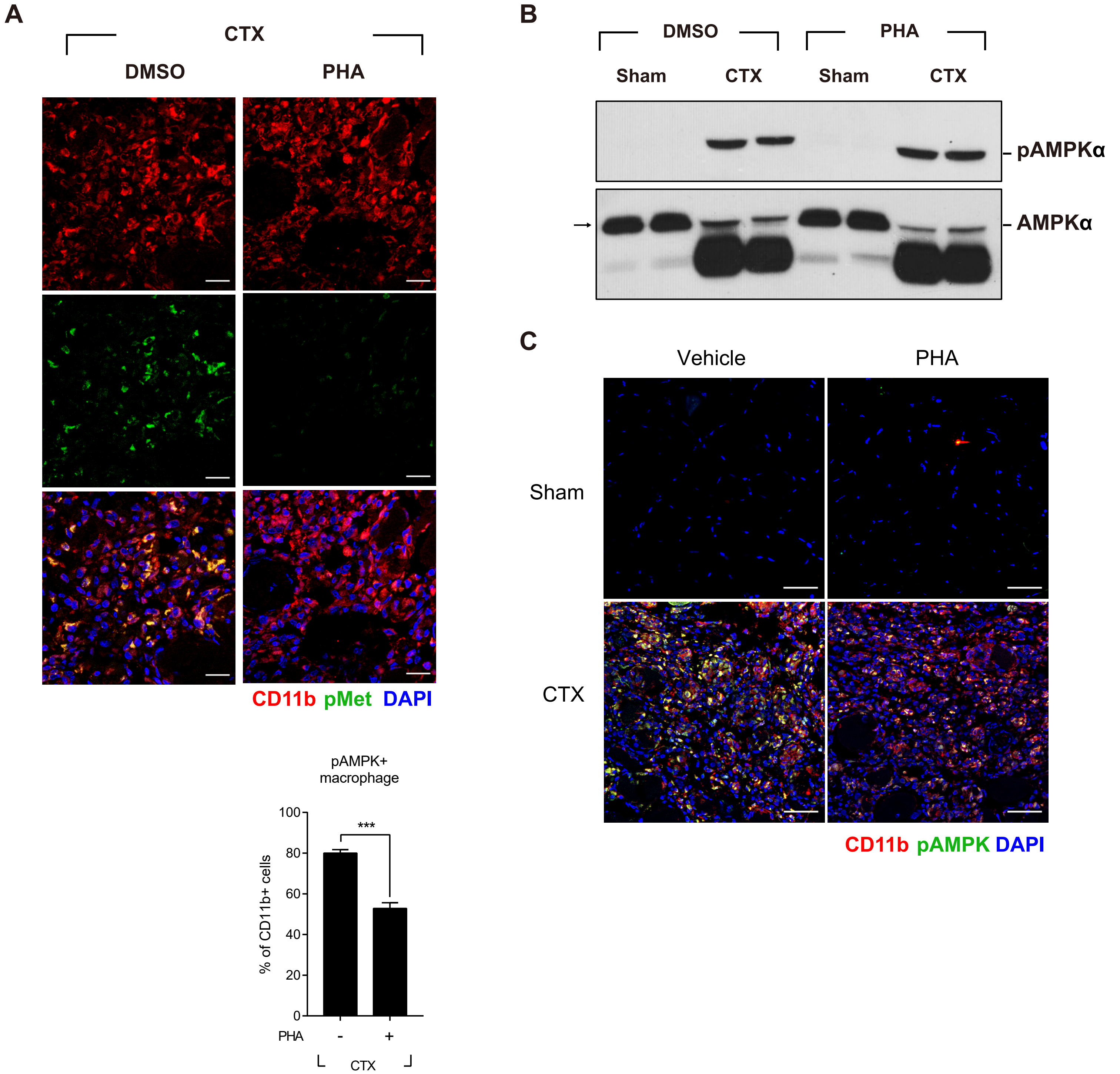

Figure S2. Effect of c-met inhibitor PHA-665752 on c-met and AMPK phosphorylation in CTX-injured muscle. After CTX injury, mice were i.p. injected with 20 mg/kg of PHA-665752 on a daily basis until sacrificed. CTX injured TAs were analyzed 3 days postinjury. (A) Effect on c-met phosphorylation in macrophages. TAs were analyzed by immunofluorescence assay using antibodies to CD11b (red), and phosphorylated c-met (green). Nuclei were counterstained with DAPI (blue). n=4 per group. Scale bars, 20 μm. (B) Effect on total and phosphorylated AMPK in TA muscle. TA muscles were prepared and total proteins were isolated followed by Western blot using antibodies to total and phosphorylated AMPK. Two representative results are shown here, n=4 per group. (C) Effect on AMPK phosphorylation in macrophages. TAs were analyzed by immunofluorescence assay using antibodies to CD11b (red), and phosphorylated AMPK (green). Nuclei were counterstained with DAPI (blue). n=4 per group. Scale bars, 50 μm. Percentage of iNOS+ or CD206+ macrophages was indicated in the graph. ***p<0.001 (unpaired student's t test)

Figure S3, related to Figure 4

A

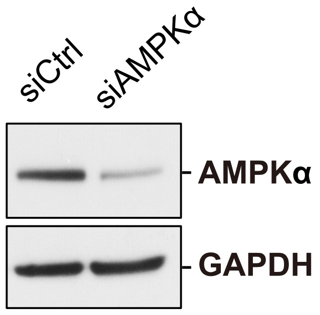

B

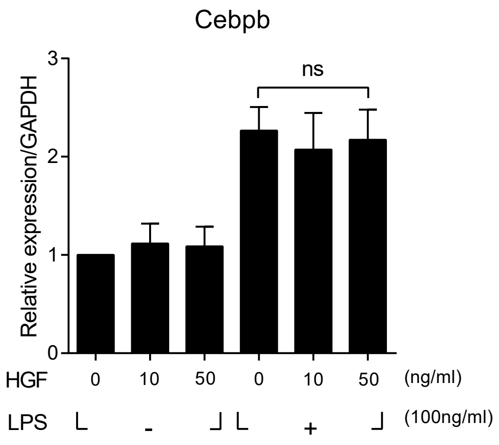

Figure S3. Effect of AMPKα knockdown and HGF-mediated regulation of cebpb expression in Raw 264.7 cells. (A) The protein level of AMPKα was determined by Western blot. Raw 264.7 cells were transfected with AMPKα or control siRNAs. Values were normalized to GAPDH. (B) Effects of HGF on the RNA level of cebpb. ns, not significant (unpaired student's t test), n=3 per group.

Figure S4, related to Figure 5

A

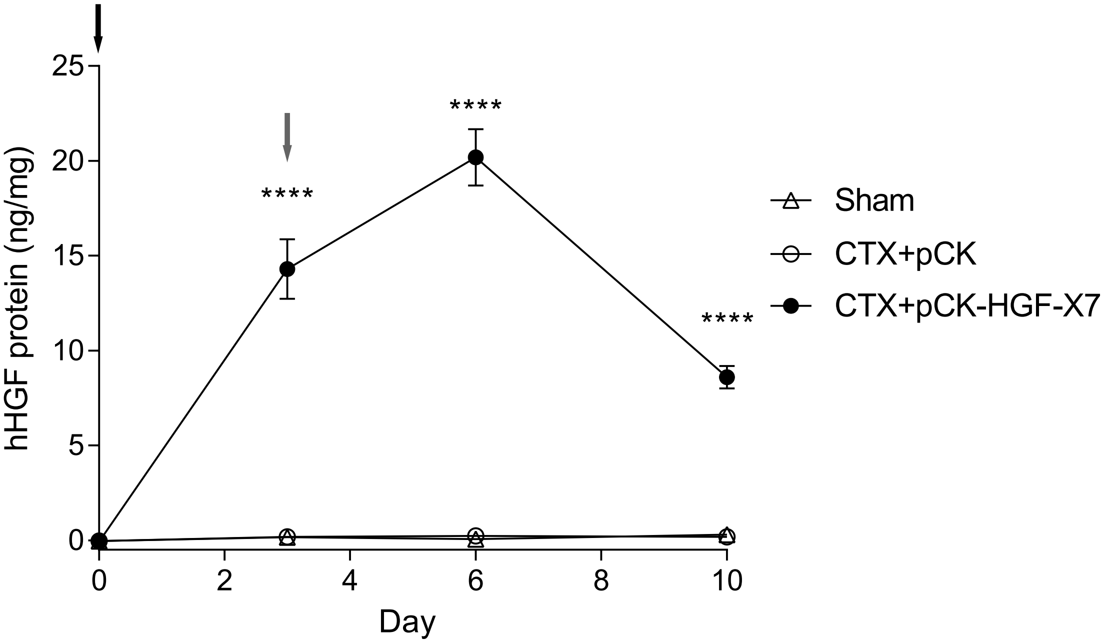

Figure S4. hHGF expression by pCK-HGF-X7 in CTX-injected mice

pCK-HGF-X7 was i.m. injected at three days prior (day 0) to the CTX injection (day 3). (A) Expression kinetics of the hHGF protein after denervation. Black arrow indicate i.m. injection of pCK-HGF-X7 on day 0 and gray arrow indicate i.m. injection of CTX on day 3. The muscle was isolated at 3, 7, and 10 days after plasmid injection, and total proteins were analyzed by ELISA to measure the protein level of human HGF. ****p<0.0001 versus pCK-treated muscle (one-way ANOVA), n=4 per group.

Table S1

A

Primers	Forward Sequence	Reverse Sequence
Hgf	5'-ATCCACGATGTTCATGAGAG-3'	5'-GCTGACTGCATTTCTCATTC-3'
IL1b	5'-CAACCAACAAGTGATATTCTCCATG-3'	5'-GATCCACACTCTCCAGCTGCA-3'
Nos2	5'-CACGGACGAGACGGATAG-3'	5'-GGGAGGAGCTGATGGAGT-3'
Ccl2	5'-TTTTGTCACCAAGCTCAAGAGA-3'	5'-ATTAAGGCATCACAGTCCGAGT-3'
IL10	5'-TACCTGGTAGAAGTGATGCC-3'	5'-CATCATGTATGCTTCTATGC-3'
Tgfb1	5'-CTCCCGTGGCTTCTAGTGC-3'	5'-GCCTTAGTTTGGACAGGATCTG-3'
Arg1	5'-AGCTCCAAGCCAAAGTCCTTAGA-3'	5'-CCTCCTCGAGGCTGTCCTT-3'
Mrc1 (CD206)	5'-CAGGTGTGGGCTCAGGTAGT-3'	5'-TGTGGTGAGCTGAAAGGTGA-3'
Chi3l3 (Ym1)	5'-GGGCATACCTTTATCCTGAG-3'	5'-CCACTGAAGTCATCCATGTC-3'
CD163	5'-TCCACACGTCCAGAACAGTC-3'	5'-CCTTGGAAACAGAGACAGGC-3'
Retnla	5'-CCCTGCTGGGATGACTGCTA-3'	5'-TGCAAGTATCTCCACTCTGGATCT-3'
Myod1	5'-CCACTCCGGGACATAGACTTG-3'	5'-AAAAGCGCAGGTCTGGTGAG-3'
Tnfa	5'-GCCACCACGCTCTTCTGTCT-3'	5'-GTCTGGGCCATGGAACTGAT-3'
Myh3	5'-CACCTGGAGAGGATGAAGAAGAA-3'	5'-AAGACTTGACTTTCACTTGGAGTTTATC-3'
Cebpb	5'-GGAGACGCAGCACAAGGT-3'	5'-AGCTGCTTGAACAAGTTCCG-3'
Gapdh	5'-CTGGAAAGCTGTGGCGTGAT-3'	5'-CCAGGCGGCACGTCAGATCC-3'

B

Antibody	Host	Company	Catalog	Notes
pMet (Y1234/1235)	rabbit	Cell Signaling	#3077	WB (1:500), IF (1:100)
Met	rabbit	Sigma Aldrich	SAB4300599	WB (1:2000)
CD11b	rat	Biolegend	101202	IF (1:200)
CD31	rat	BD Biosciences	550274	IF (1:200)
Ly6G	rat	Abcam	ab25377	IF (1:200)
iNOS	rabbit	Millipore	06-573	IF (1:200)
CD206	rabbit	Abcam	ab64673	IF (1:200)
MyoD	rabbit	Santacruz	sc-304	WB (1:500)
pLKB1 (S428)	rabbit	Cell Signaling	#3482	WB (1:1000)
LKB1	rabbit	Cell Signaling	#3047	WB (1:1000)
pCREB (S133)	rabbit	Cell Signaling	#9198	WB (1:1000)
CREB	rabbit	Cell Signaling	#4820	WB (1:1000)
pAMPKα (T172)	rabbit	Cell Signaling	#2535	WB (1:1000)
AMPKα	mouse	Cell Signaling	#2793	WB (1:1000)
pCaMKKβ (S511)	rabbit	Cell Signaling	#12818	WB (1:1000)
CaMKKβ	rabbit	Abcam	ab124096	WB (1:500)
Myh3	mouse	DSHB	BF-45	IF (1:50)
β-tubulin	rabbit	Abcam	ab6046	WB (1:1000)
GAPDH	mouse	Abcam	ab8245	WB (1:5000)

Table S1. Primers and antibodies used in this study.
(A) Nucleotide sequences of primers used for RT-qPCR analysis. (B) Antibodies used for Western blot hybridization and Immunofluorescence.
